# Increased functional connectivity between brain regions involved in social cognition, emotion and affective-value in psychedelic states induced by N,N-Dimethyltryptamine (DMT)

**DOI:** 10.3389/fphar.2024.1454628

**Published:** 2024-10-30

**Authors:** Carla Soares, Gisela Lima, Marta Lapo Pais, Marta Teixeira, Célia Cabral, Miguel Castelo-Branco

**Affiliations:** ^1^ Coimbra Institute for Biomedical Imaging and Translational Research (CIBIT), Institute of Nuclear Sciences Applied to Health (ICNAS), University of Coimbra, Coimbra, Portugal; ^2^ Faculty of Medicine (FMUC), University of Coimbra, Coimbra, Portugal; ^3^ Clinic Academic Center of Coimbra (CACC), Coimbra Institute for Clinical and Biomedical Research (iCBR), Faculty of Medicine, University of Coimbra, Coimbra, Portugal; ^4^ Center for Innovative Biomedicine and Biotechnology (CIBB), University of Coimbra, Coimbra, Portugal; ^5^ Department of Life Sciences, Centre for Functional Ecology, University of Coimbra, Coimbra, Portugal

**Keywords:** psychedelics, N,N-Dimethyltryptamine (DMT), pharmacoimaging, functional connectivity, social cognition, fMRI

## Abstract

The modulation of social cognition is suggested as a possible mechanism contributing to the potential clinical efficacy of psychedelics in disorders involving socio-emotional and reward processing deficits. Resting-state functional Magnetic Resonance Imaging (rs-fMRI) can be used to detect changes in brain connectivity during psychedelic-induced states. Thus, this pharmacoimaging study investigates the effects of N,N-Dimethyltryptamine (DMT) on functional connectivity in brain areas relevant to social cognition, using a within-subject design in eleven healthy experienced users. The study included both an active and a control condition, conducted at different time points. The active condition involved DMT inhalation, while the control condition did not. Seed-based connectivity was measured for the two core regions involved in theory of mind and emotional processing, respectively, the posterior supramarginal gyrus and the amygdala. DMT increased supramarginal gyrus connectivity with the precuneus, posterior cingulate gyrus, amygdala, and orbitofrontal cortex. Additionally, increased connectivity emerged between the amygdala and orbitofrontal cortex. These results demonstrate that DMT modulates brain connectivity in socio-emotional and affective-value circuits, advancing our understanding of the neural mechanisms underlying the psychedelic experience and its potential therapeutic action.

## 1 Introduction

Adaptive social interactions rely on our ability to accurately perceive and interpret socially relevant information, understand others’ thoughts and feelings, and respond appropriately - all of which are aspects of social cognition (Beaudoin and Beauchamp, 2020). Navigating social contexts relies on the interplay between automatic and more deliberative processes generally associated with emotion perception and empathy, as well as those connected to the theory of mind and moral processing (Adolphs, 2009; Li et al., 2014). A set of cortical and subcortical brain regions, along with their interconnecting pathways collectively contribute to social cognition processes. The neural basis of emotion processing and empathy is routed in core regions such as the amygdala, ventromedial and orbitofrontal cortex, insula, and anterior cingulate cortex, allowing one to detect socially salient stimuli, such as facial expressions, evaluate their affective value, and remain emotionally engaged in social interactions (Adolphs, 2002; Adolphs, 2009; Han et al., 2021). Concurrently, the theory of mind (ToM)/mentalizing network, associated with the dorsomedial prefrontal cortex, temporoparietal junction (TPJ), and posterior cingulate gyrus, enables one to infer the mental states of others, distinguishing them from our own (Molenberghs et al., 2016; Schurz et al., 2014). Although not specific to social cognition, these brain regions and networks enable the regulation of social behavior, which is a fundamental element of mental health.

Atypical activity and connectivity within the “social brain” networks have been observed in conditions such as Autism Spectrum Disorders (ASD) (Müller and Fishman, 2018; Sato and Uono, 2019; von dem Hagen et al., 2013). Although social cognitive impairments have been more commonly associated with autism and schizophrenia, recent meta-analyses suggest they are a cognitive phenotype present in many developmental, neurological, and psychiatric conditions (Cotter et al., 2018). Deficits in emotion recognition, perspective-taking, and social reward processing have been observed in conditions such as depression and addiction (Quednow, 2020; Solomonov et al., 2023; Weightman et al., 2014).Nevertheless, there is currently a lack of effective pharmacological treatments addressing social cognition deficits (Patin and Hurlemann, 2015; Riccardi et al., 2021). Psychedelics have seen a resurgence of interest as potential treatments for these conditions, and there is evidence suggesting that this may be related to their impact on social cognition (Vollenweider and Preller, 2020). Previous studies have shown that psychedelics can modulate social processing by altering the recognition of emotions in facial expressions (Rocha et al., 2019), joint attention during social interactions (Preller et al., 2018), and empathy (Dolder et al., 2016; Mason et al., 2019). Furthermore, feelings of connectedness with other people have also been described in the psychedelic experience (van Elk and Yaden, 2022). Despite the available evidence, the mechanisms by which psychedelics affect social cognition networks are not yet fully understood.

The tryptamine psychedelic N,N-Dimethyltryptamine (DMT) is a naturally occurring compound found in several plant species and animals (Carbonaro and Gatch, 2016). It is known for its intense and relatively short-lived effects on perception, cognition, and mood, including visual imagery and mystical-type experiences (Strassman, 1996; Vogt et al., 2023). Currently, there is interest in the therapeutic potential of intravenous DMT, with clinical trials investigating its safety and efficacy in individuals with Major Depressive Disorder (D’Souza et al., 2022; Good et al., 2023). The neural correlates of intravenously administered DMT, as observed using fMRI, showed significant decreases in the within-network integrity of resting-state networks and increases in global functional connectivity. These changes were correlated with reduced power of alpha oscillations and increased entropy in EEG measurements (Timmermann et al., 2023). Additionally, DMT-induced states are characterized by heightened temporal lobe activity alongside medial parietal and hippocampal hypoactivity, with the latter being associated with both the meaningfulness of the psychedelic experience and variations in heart rate (Pasquini et al., 2024).

While there is increasing interest in the study of intravenously administered DMT, the effects of inhaled DMT on the brain remain poorly understood. A previous study reported that DMT inhalation in natural settings leads to decreased power of alpha oscillations and increased power of delta and gamma oscillations (Pallavicini et al., 2021). However, the underlying BOLD (Blood Oxygenation Level Dependent) signal fluctuations at a higher spatial resolution through this route of administration are not known.

Meta-analytic evidence from functional imaging studies focusing on the effects of tryptamine psychedelics in brain activity and connectivity has indicated their impact in brain regions associated with theory of mind and affective regulation (Castelhano et al., 2021). Specifically, the study highlighted a robust neuromodulatory effect in the amygdala and identified a region within the temporoparietal junction (TPJ), namely, the supramarginal gyrus (Castelhano et al., 2021). Additionally, a conjunction analysis between brain activations during social cognition tasks and psychedelic-induced states demonstrated the supramarginal gyrus as a region of joint activation (Soares et al., 2023). Connectivity in the pSMG is crucial for attention reorientation, an essential aspect of social cognition that involves understanding surroundings and switching between self- and other-related representations (Quesque and Brass, 2019). This region is implicated in the self-other distinction within the emotional domain (Riva et al., 2022), and its preserved volume is associated to emotion recognition abilities (Wada et al., 2021). Although not a primary hub, the amygdala also significantly influences social processes. Amygdala lesions have been associated with impairments in social behavior, including difficulties in recognizing and responding to social stimuli, and challenges in theory of mind abilities (Meisner et al., 2022). The amygdala’s circuits, particularly its connections with the medial prefrontal cortex (mPFC) and orbitofrontal cortex (OFC) are crucial for social perception, decision-making and social interaction (Meisner et al., 2022), as well as for emotional regulation, which is essential for adaptive social interactions. Both the pSMG in the mentalizing network and the amygdala have been shown to be modulated by psychedelics, suggesting that their connectivity patterns may help in understanding the impact of psychedelics on socio-emotional domains. Consequently, we investigated the effects of inhaled DMT on resting-state functional connectivity, with a particular focus on the core roles of the posterior supramarginal gyrus (pSMG) within the TPJ and the amygdala, as key hubs of the social cognition and affective networks.

Drawn from recent models of the neural basis of psychedelic action proposing an increased global functional connectivity and disintegration of association networks (Kwan et al., 2022), we hypothesized increased functional connectivity between areas of the mentalizing network and limbic/reward areas. If DMT alters the functional connectivity of social cognition networks, this could have therapeutic implications for clinical populations with altered functional connectivity patterns, such as autism spectrum disorder. In light of these considerations, our experimental design involved the self-administration of a DMT-based extract by experienced users, aligned with their typical patterns of use. Immediately following the inhalation, resting-state fMRI data were collected to capture brain connectivity during the peak phase of DMT’s effects. By examining the pSMG and amygdala, our study seeks to elucidate how DMT influences neural circuits involved in social cognition and affect. Consequently, the findings may offer insights into the acute effects of DMT and its potential therapeutic applications for disorders characterized by socio-emotional deficits.

## 2 Methods

### 2.1 Study design

We used a within-subject design consisting of active and control conditions separated by an interval of 4–6 weeks. Since participants served as their own controls, confounding variables were minimized, thereby enhancing statistical power by reducing individual variability. In the active condition (DMT condition), individuals inhaled the substance immediately before fMRI acquisition, following their usual ritualistic procedures. A specially decorated, comfortable room was arranged for participants to prepare for the experience and to stay afterward. All volunteers were experienced DMT users who engaged in their customary rituals before inhalation, incorporating elements such as setting intentions for the experience, meditation, chant singing, drumming, symbolic actions, and the burning of plants such as Palo Santo and white sage. Following this preparation, participants inhaled a DMT extract through a pipe, consistent with their typical patterns of use. This procedure occurred in an outside area in close proximity (a few meters) to the MRI room door, ensuring minimal delay between substance administration and imaging. Participants had the option to inhale while seated and then be instantly transported to the MRI machine in a wheelchair, or to walk directly to the MRI machine on foot, which took about 1 min. A total of 4 min passed between the inhalation and the initiation of the first fMRI acquisition sequence. Our study refers to the effects occurring after this time window, which does not capture the very onset of the DMT effects. In the control condition, the same rituals and preparations for the psychedelic experience were conducted without substance administration. The inhaled DMT-based extract [root bark of *Mimosa hostilis* Benth., a synonym of *Mimosa tenuiflora* (Willd.) Poir.] was produced with the help of an expert participant, with all doses sourced from a single batch and weighed before administration to ensure consistency. Participants inhaled approximately 50–70 mg of the DMT extract, with quantities adjusted to account for individual variations in the combustion and inhalation processes.

The DMT present in the sample was quantified by high-performance liquid chromatography (HPLC-DAD) revealing a percentage of 30.92% DMT. For detailed information on DMT quantification, please see Pais et al. (2024).

Resting-state functional Magnetic Resonance Imaging (rs-fMRI) scans were first acquired after the participants inhaled the DMT. The data analysed in this paper were collected as part of a larger project, involving other fMRI tasks that will be reported in other studies.

A team comprising a medical doctor and two psychologists supported the participants throughout all stages of the protocol. The study was approved by the Ethics Committee of the Faculty of Medicine of the University of Coimbra and conducted in accordance with the principles of the Declaration of Helsinki. All participants provided written informed consent prior to participating in the study.

### 2.2 Participants

The study enrolled 11 healthy adult volunteers, including 4 females and 7 males, with a mean age of 37 ± 12.4 years. Socioeconomic backgrounds were categorized as medium (N = 3) and medium-high (N = 8), and participants had completed either a university degree (N = 8) or high school (N = 3). In terms of professional status, the group included students (N = 2), employed individuals (N = 5), unemployed individuals (N = 3), and one retired participant (N = 1). All volunteers had substantial experience with DMT/changa inhalation (more than 10 times) and had prior use of other psychoactive substances, such as LSD, MDMA, psilocybin, and cannabis. Recruitment initially involved social media advertisements and word-of-mouth. Included participants referred additional potential volunteers, and an online screening interview was conducted before their inclusion in the study. The Mini International Neuropsychiatric Interview (MINI) (Lecrubier et al., 1992; Amorim, 2020; Sheenhan et al., 1998) and the Mini Mental State Examination (MMSE) (Santana et al., 2016) were used to exclude volunteers with current psychiatric and neurological disorders. Participants with first-degree relatives with schizophrenia, bipolar disorder, or mania/hypomania were also excluded. Some participants (N = 4) had a history of mild, non-diagnosed depression but had been symptom-free for at least 5 years. Additional exclusion criteria included individuals with cardiac or liver diseases; immunosuppression; use of antihypertensive, sympathomimetic, antidepressant, or benzodiazepine drugs; being a member of the project team; and pregnancy. The inclusion criteria required participants to have prior experience with self-administration of DMT (at least 5 DMT/changa sessions). Out of the 13 participants screened, one withdrew from the study and another was excluded, resulting in 11 participants in the final analysis. Participants were instructed to abstain from psychoactive substances for 2 months before the study sessions. Regarding caffeine and tobacco, participants were asked to abstain for 24 h.

### 2.3 Imaging

The MRI scans were acquired using a 3T imaging system (MAGNETOM Prisma, Siemens Medical Solutions) with a 64-channel head-coil. We collected resting-state functional images using a 7-min sequence, and a total of 210 volumes. Participants were instructed to close their eyes during this period to minimize any interference with their psychedelic experience. Resting-state BOLD fMRI scans had the following parameters: Repetition Time (TR) = 2000 ms; Echo Time (TE) = 20 ms; flip angle = 82°; slices = 50 of thickness = 2.5 mm; field of view (FOV) = 195 mm × 195 mm; voxel size = 2.5 mm × 2.5 mm × 2.5 mm. Additionally, we acquired T1-weighted anatomical MRI data at a spatial resolution of 1 × 1 × 1 mm^3^, with a TR of 2530 ms, TE of 3.5 ms, and a Flip Angle of 7°.

### 2.4 Assessment of psychedelic experience

The Hallucinogen Rating Scale (HRS) (Strassman et al., 1994) was used to measure the subjective effects of DMT administration. The HRS is a 100-item questionnaire that employs a 0–4 rating scale to evaluate the subjective effects of psychedelic substances across six domains: somaesthesia, affect, perception, cognition, volition and intensity. The HRS was administered after the MRI procedure, at a point when participants felt prepared to communicate their experiences.

### 2.5 Data analysis

Resting-state functional images were analysed using CONN (Whitfield-Gabrieli and Nieto-Castanon, 2012) (RRID:SCR_009550) release 20.b (Nieto-Castanon and Whitfield-Gabrieli, 2022) and SPM (RRID:SCR_007037) release 12.7771 (Penny et al., 2011).

Functional and anatomical data were preprocessed using a flexible preprocessing pipeline (Nieto-Castanon, 2020) including realignment with correction of susceptibility distortion interactions, slice timing correction, outlier detection, direct segmentation and MNI-space normalization, and smoothing. Functional data were realigned using SPM realign and unwarp procedure (Andersson et al., 2001), where all scans were coregistered to a reference image (first scan of the first session) using a least squares approach and a 6 parameter (rigid body) transformation (Friston et al., 1995), and resampled using b-spline interpolation to correct for motion and magnetic susceptibility interactions. Temporal misalignment between different slices of the functional data (acquired in interleaved Siemens order) was corrected following SPM slice-timing correction procedure (Sladky et al., 2011), using sinc temporal interpolation to resample each slice BOLD timeseries to a common mid-acquisition time. Potential outlier scans were identified using ART as acquisitions with framewise displacement above 0.5 mm or global BOLD signal changes above 3 standard deviations (Power et al., 2014), and a reference BOLD image was computed for each subject by averaging all scans excluding outliers. Functional and anatomical data were normalized into standard MNI space, segmented into grey matter, white matter, and CSF tissue classes, and resampled to 2 mm isotropic voxels following a direct normalization procedure (Calhoun et al., 2017) using SPM unified segmentation and normalization algorithm (Ashburner, 2007; Ashburner and Friston, 2005) with the default IXI-549 tissue probability map template. Last, functional data were smoothed using spatial convolution with a Gaussian kernel of 8 mm full width half maximum (FWHM).

In addition, functional data were denoised using a standard denoising pipeline (Nieto-Castanon, 2020) including the regression of potential confounding effects characterized by white matter timeseries (10 CompCor noise components), CSF timeseries (5 CompCor noise components), motion parameters and their first order derivatives (12 factors) (Friston et al., 1996), outlier scans (below 182 factors) (Power et al., 2014), and linear trends (2 factors) within each functional run, followed by high-pass frequency filtering of the BOLD timeseries (Hallquist et al., 2013) above 0.01 Hz. CompCor (Behzadi et al., 2007; Chai et al., 2012) noise components within white matter and CSF were estimated by computing the average BOLD signal as well as the largest principal components orthogonal to the BOLD average, motion parameters, and outlier scans within each subject’s eroded segmentation masks.

Concerning the first-level analysis, seed-based connectivity maps (SBC) were estimated characterizing the spatial pattern of functional connectivity with a seed area. Two seed regions were selected for their significance as core regions in social and emotional networks: the posterior segment of the supramarginal gyrus (pSMG) within the temporoparietal junction (TPJ) and the amygdala in both the right and left hemispheres. To define these seed regions, we used the default atlas provided by the CONN toolbox, which combines cortical and subcortical areas from the FSL Harvard-Oxford atlas (Frazier et al., 2005; Makris et al., 2006; Desikan et al., 2006; Goldstein et al., 2007) with cerebellar areas from the AAL atlas (Tzourio-Mazoyer et al., 2002). Functional connectivity strength was represented by Fisher-transformed bivariate correlation coefficients from a weighted general linear model (weighted-GLM), estimated separately for each seed area and target voxel, modeling the association between their BOLD signal timeseries. Individual scans were weighted by a boxcar signal characterizing each experimental condition convolved with an SPM canonical hemodynamic response function and rectified.

Group-level analyses were performed using a General Linear Model (GLM). For each individual voxel a separate GLM was estimated, with first-level connectivity measures at this voxel as dependent variables and groups as independent variables. Voxel-level hypotheses were evaluated using multivariate parametric statistics with random-effects across subjects and sample covariance estimation across multiple measurements. We examined the alterations in functional connectivity resulting from DMT administration, focusing on both increases and decreases. This was performed using a seed-to-voxel analysis with a between-conditions contrast (DMT > Control) assessed through a paired *t*-test. Given the absence of a lateralization hypothesis for each selected seed, we performed an F-test to assess the conjunction analysis across both the right and left hemispheres. Inferences were performed at the level of individual clusters. Cluster-level inferences were based on parametric statistics from Gaussian Random Field theory (Worsley et al., 1996). Results were thresholded using a combination of a cluster-forming *p* < 0.001 voxel-level threshold, and a familywise corrected p-FDR<0.05 cluster-size threshold (Chumbley et al., 2010).

The self-report data from the Hallucinogen Rating Scale (HRS) were analyzed using SPSS (version 28.0.0.0). A repeated-measures ANOVA was performed, with the within-factor of condition (DMT vs Control) as the main variable of interest. The significance threshold was set at *p* < 0.05. Lastly, to explore the relationship between the imaging and self-report data, Spearman’s correlation analysis was performed. Initially, we computed the difference in connectivity (Δr) between the DMT and control conditions. Additionally, we determined the difference in scores of the Hallucinogen Rating Scale (ΔHRS) between both conditions. Finally, we investigated the correlation between the previously calculated Δr and ΔHRS between conditions, employing a significance threshold of *p* < 0.05.

## 3 Results

**FIGURE 1:**
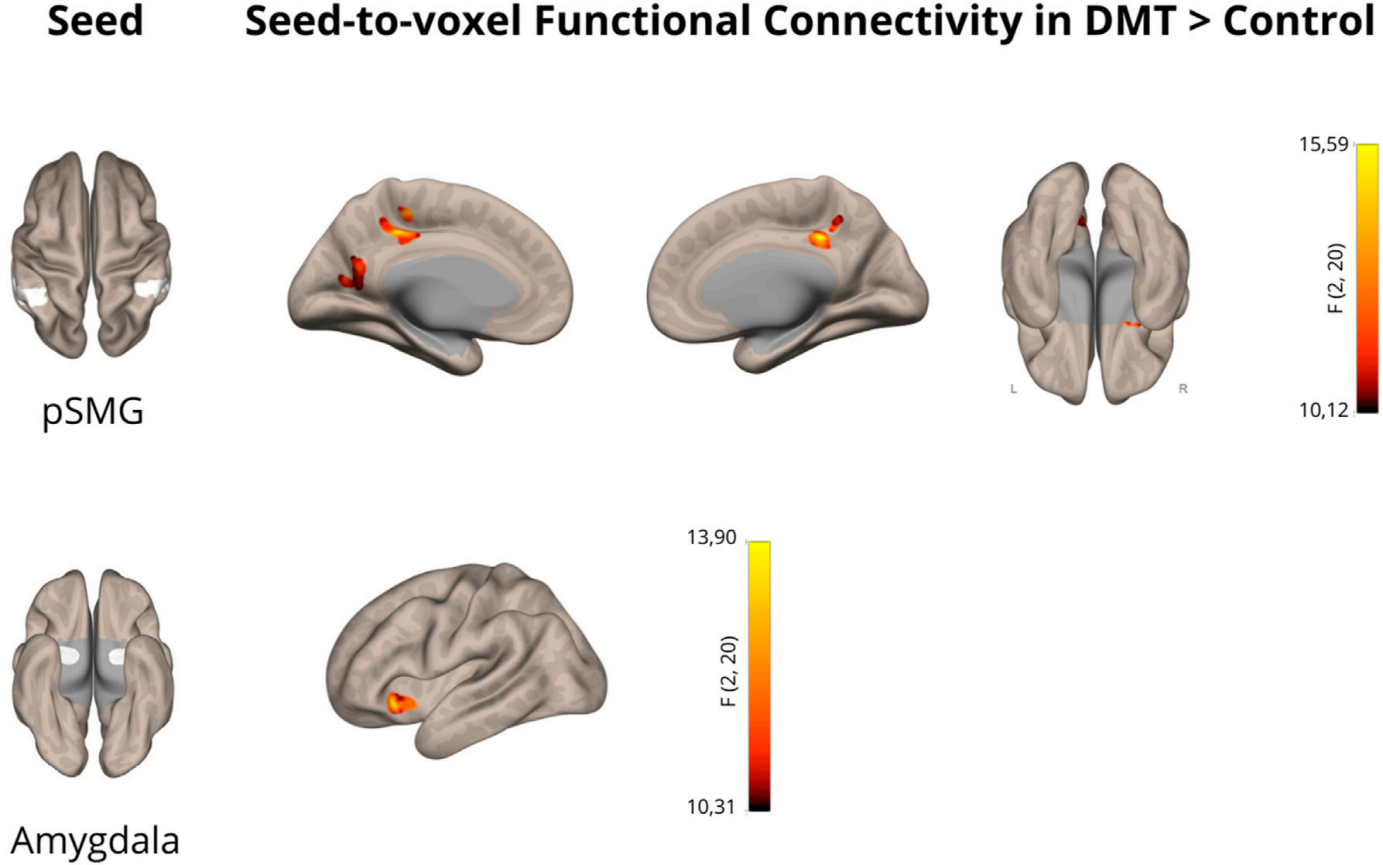
N,N-Dimethyltryptamine (DMT) increases functional connectivity in the posterior supramarginal gyrus and amygdala. Cortical maps showing significant alterations in functional connectivity using the posterior supramarginal gyrus (pSMG) and the amygdala as seeds in the DMT > Control contrast (*p* < 0.05 cluster-size p-FDR corrected). The color bar represents the joint effects across both hemispheres for the selected seeds.

In this pharmacoimaging study, we investigated the effect of DMT on resting-state functional connectivity within brain regions associated with social cognition in a group of 11 healthy volunteers. Compared to the control condition, DMT administration significantly increased supramarginal gyrus connectivity with three clusters, namely, the posterior cingulate gyrus (*p* < 0.05, FDR corrected), the precuneus (*p* < 0.05, FDR corrected) and a cluster encompassing the right amygdala and orbitofrontal cortex (*p* < 0.05, FDR corrected). Detailed findings are presented in Table 1 and depicted in Figure 1. These findings provide further evidence supporting the impact of DMT on increased connectivity between the temporoparietal junction, representing the core of social cognition, and limbic and reward brain areas.

**TABLE 1:** Enhanced seed-based connectivity during DMT (vs. Control)**.** Results of functional connectivity analysis using two seeds: the posterior supramarginal gyrus (pSMG); and the amygdala. Montreal Neurological Institute (MNI) coordinates represent peak locations of significantly increased connectivity with other brain regions. Results are shown at an FDR-corrected cluster-level threshold of *p* < 0.05.

Peak cluster (x, y, z)			Hemisphere	Size (k)	Brain region	p-FDR corrected
Seed: Posterior Supramarginal Gyrus (pSMG)						
+06	−34	+38	R/L	338	Posterior Cingulate Gyrus	0.000313
−04	−62	+18	R/L	114	Precuneus	0.048600
+20	+06	−16	R	114	Amygdala/Orbitofrontal Cortex	0.048600
Seed: Amygdala						
−34	+22	−10	L	128	Orbitofrontal Cortex	0.025213

Furthermore, DMT administration resulted in increased amygdala functional connectivity with the left orbitofrontal cortex (*p* < 0.05, FDR corrected), as shown in Table 1 and Figure 1, further indicating the impact of DMT on emotion and reward related brain networks.

**FIGURE 2:**
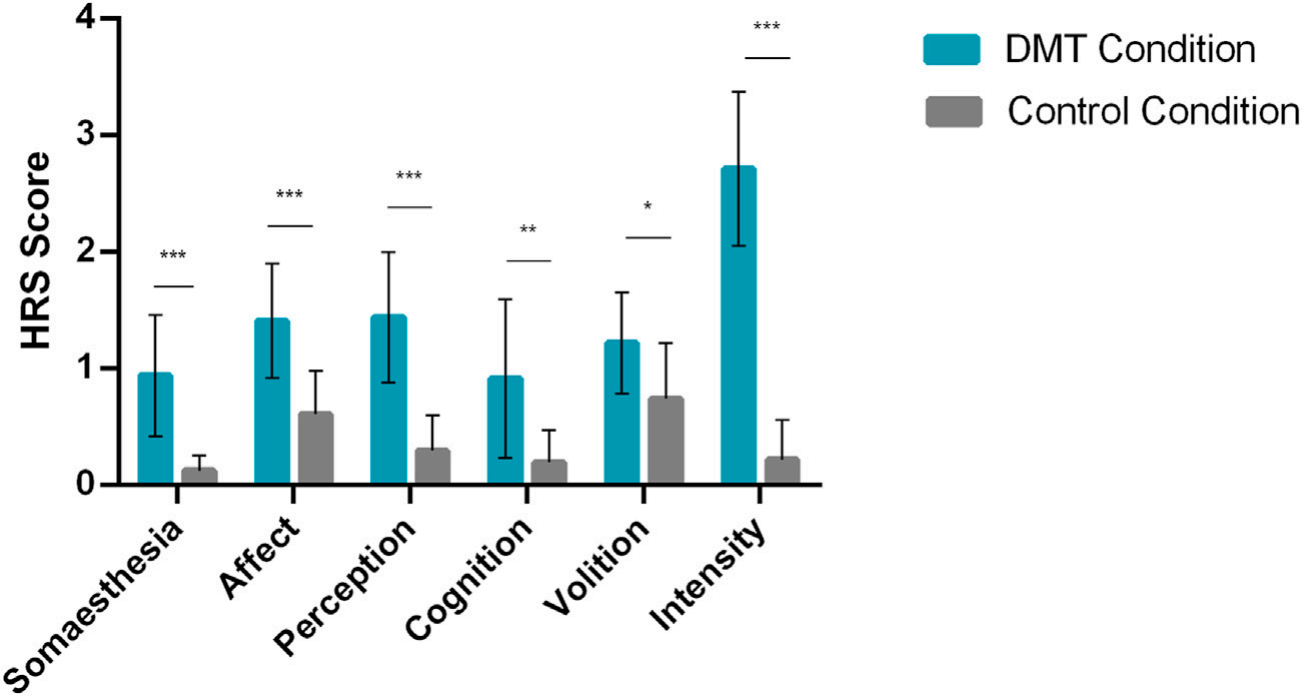
Psychedelics effects: N,N-Dimethyltryptamine (DMT) significantly increases Hallucinogen Rating Scale (HRS) scores. Bars show mean HRS scores for DMT and Control conditions, error bars show standard deviation (N = 11). Participants reported significantly higher HRS scores in the DMT condition. Asterisks correspond to the following significance levels: *p < 0.05; **p < 0.01 and ***p < 0.001.

The inhalation of DMT also produced several subjective effects. We found significant differences between conditions for all HRS scales, including increases in somaesthesia (*p* < 0.001), affect (*p* < 0.001), perception (*p* < 0.001), cognition (*p* < 0.01), volition (*p* < 0.05), and intensity (*p* < 0.001) in the DMT condition compared to placebo, as shown in Figure 2. The detailed information regarding means and effect sizes can be found in Supplementary Table S1. No adverse effects were observed.

**FIGURE 3:**
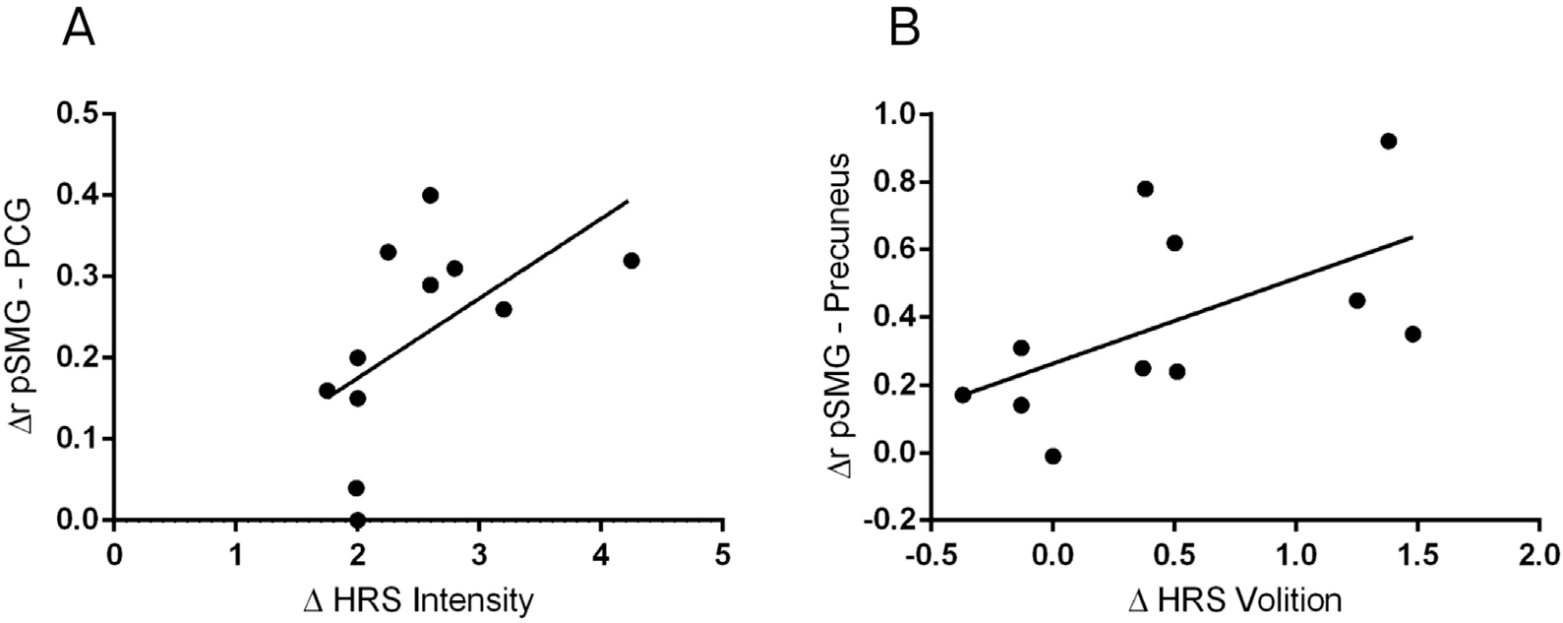
Correlations between DMT-induced changes in functional connectivity and psychedelic effects. Significant Spearman’s correlation between functional connectivity changes between the posterior supramarginal gyrus and the posterior cingulate gyrus (∆ pSMG - PCG) and the increases in the Intensity Scale of the Hallucinogen Rating Scale (HRS) **(A)**. Significant Spearman’s correlation between changes in pSMG–Precuneus functional connectivity and the HRS Volition Scale **(B)**.

Spearman’s correlation was employed to investigate the relationship between functional connectivity and the Hallucinogen Rating Scale (HRS). The differences between conditions in each scale were tested alongside significant changes in functional connectivity of the selected seeds. Figure 3 presents the significant correlations between ∆r and ∆HRS. The findings highlight a relationship between altered brain connectivity and self-reported psychedelic effects. Specifically, a positive correlation was observed between changes in functional connectivity between the pSMG and posterior cingulate gyrus and the increases in intensity effects, r_s_ (9) = 0.70, *p* < 0.05, as illustrated in Figure 3A. Additionally, a positive correlation was also found between changes in the functional connectivity between the pSMG and the precuneus and the volition effects, r_s_ (9) = 0.62, *p* < 0.05 (Figure 3B). However, these correlations did not survive multiple comparisons correction. Supplementary Table S2 presents detailed information on ∆r, ∆HRS, and r coefficients. No significant correlations were found for the other subscales (all *p* > 0.05). These results suggest the interplay between neural mechanisms and the subjective experience during DMT-induced states. It is also worth noting that there was a significant correlation between the connectivity of the amygdala and orbitofrontal cortex with the Affect Scale during the DMT condition (r (9) = -0.607, *p* = 0.048), although the direct comparison with the Control condition did not reach statistical significance.

## 4 Discussion

Our results provide evidence corroborating our hypothesis that DMT induces changes in functional connectivity within brain regions relevant to social cognition and in regions associated with emotion and affective value. Moreover, the changes in connectivity strength were correlated with increases in self-reported psychedelic effects, particularly in intensity and volition. These correlations highlight the intricate relationships between the neural effects of DMT in socioemotional circuits and subjective experiences.

The impact of DMT on subjective experience led to an increase in the intensity of effects, along with changes in somaesthesia, affect, perception, cognition, and volition. Typically, we would expect an increase in all these HRS scales under the DMT condition, except for volition (Riba et al., 2001; Strassman et al., 1996). Contrary to expectations, volition scores increased, a pattern also observed in other studies (Palhano-Fontes et al., 2019; Riba et al., 2006). This result may be related to factors such as the participants’ previous experience with psychedelics, the nature of the DMT experience, and the supportive environment in which the study was conducted. These factors could enhance feelings of agency and control, even during intense psychedelic experiences, which could be related to the effect of DMT on functional connectivity. We observed significant enhancements in functional connectivity between the posterior supramarginal gyrus, the posterior cingulate gyrus and the precuneus, all of which play key roles in the theory of mind (ToM) network, a fundamental aspect of social cognition (Schurz et al., 2014). Additionally, alterations in functional connectivity were evident in the amygdala and orbitofrontal cortex, both of which have also been linked to the affective domain of the ToM (Abu-Akel and Shamay-Tsoory, 2011). The right supramarginal gyrus plays a role in mitigating the emotional egocentricity bias in empathic judgments, facilitating an effective self-other distinction within the emotional domain (Silani et al., 2013). Together with cingulate regions, it is implicated in evaluating emotions and pain in others (Corradi-Dell’Acqua et al., 2014). These neural mechanisms can be related to the reported effects of psychedelics on increased states of empathy (Kiraga et al., 2021; Uthaug et al., 2021 Dolder et al., 2016; Mason et al., 2019).

Moreover, increased functional connectivity was found between the posterior supramarginal gyrus and the right amygdala and orbitofrontal cortex. This suggests that DMT influences the connections between the temporoparietal region and the limbic and reward systems. Recent advances in social neuroscience research have shifted the focus from studying the mere perception of social stimuli to more ecological social interaction paradigms (Redcay and Schilbach, 2019). The results suggest that engaging in social interactions, rather than solely mentalizing about others’ mental states, recruits the theory of mind and reward networks, supporting the notion that their interplay facilitates social behaviour (Alkire et al., 2018; Redcay and Schilbach, 2019; Warnell et al., 2018). The observed increase in connectivity between areas of both networks under the influence of DMT may shed light on the impact of psychedelics on social behaviour. Preclinical evidence has provided insights into psychedelics’ ability to reopen critical periods for social reward learning, potentially holding implications for disorders such as autism (Nardou et al., 2023). These critical periods represent windows of time in which the brain becomes highly sensitive to stimuli and adaptable to synaptic, circuit and behavioural changes. According to the authors, this occurs since psychedelics interact with binding targets, including Serotonin Transporter (SERT), 5-Hydroxytryptamine 2A Receptor (5-HT_2A_R), N-Methyl-D-aspartate Receptor (NMDA), Kappa Opioid Receptor (KOR), triggering the degradation of the extracellular matrix (ECM), which, in turn, enables metaplastic processes. By promoting metaplasticity, psychedelics can open critical periods lasting for several weeks, which may underlie the therapeutic potential of psychedelics by fostering psychological flexibility and cognitive reappraisal (Nardou et al., 2023). These collective findings warrant further investigation into the impact of psychedelics on the “social brain” and behaviour.

In addition, we found that the administration of DMT led to increased functional connectivity between the amygdala and the left orbitofrontal cortex. These regions are well-established for their roles in emotion processing and the subjective experience of affective value (Rolls et al., 2020). Within the social domain, the amygdala-orbitofrontal circuit is associated with linking face perception to emotion understanding, and the processing of value underlying social interactions (Adolphs, 2002; Meisner et al., 2022). Reduced functional connectivity between the amygdala and orbitofrontal cortex has been reported in anxiety (Lindner et al., 2018), social anxiety (Hahn et al., 2011), depression (Rolls et al., 2020), as well as in youths with psychopathic traits during moral judgment tasks (Marsh et al., 2011). This pathway is therefore instrumental for emotion regulation. The tendency of a negative correlation between amygdala-OFC connectivity and the HRS affect scale suggests that higher connectivity between these regions is associated with fewer emotional changes, potentially indicating enhanced emotional control during the psychedelic experience. This finding might elucidate previous evidence showing reduced amygdala activity during the recognition of fearful faces following LSD and psilocybin administration (Kraehenmann et al., 2015; Mueller et al., 2017). Increased amygdala-OFC connectivity may be a key circuit for understanding how negative stimuli can be processed without emotional dysregulation. Moreover, meta-analytic evidence revealed the ability of psychedelics to modulate the amygdala activity (Castelhano et al., 2021). These findings suggest that the fronto-amygdalar connectivity could represent a mechanism of interest in understanding the potential therapeutic effects of psychedelics.

Previous studies have reported the deactivation of the Default Mode Network (DMN) under psychedelic states (Kwan et al., 2022; van Elk and Yaden, 2022), a network that exhibits a large overlap with the brain regions involved in social cognition. Despite multiple measures of connectivity and preprocessing making cross-study comparisons difficult (McCulloch et al., 2022), our study puts in new perspective prior deactivation findings of DMN induced by psychedelics (Kwan et al., 2022), suggesting that increased connectivity may be more important than changes in activity levels. Accordingly, our results align with data-driven approaches demonstrating that psychedelics increase global functional connectivity in the temporoparietal junction (Tagliazucchi et al., 2016). These changes in connectivity correlated with subjective reports of ego dissolution, implying a reduced differentiation between the self and others (Tagliazucchi et al., 2016). Additionally, evidence shows that LSD alters self-processing during a social interaction task, accompanied by reduced activity of the posterior cingulate cortex and the angular gyrus. Collectively, these findings suggest that psychedelics modify self and others’ perception, thereby modulating social cognition. By contributing to these previous findings, our study shows that DMT modulates the functional connectivity between the theory of mind, limbic, and reward brain areas, raising the hypothesis that the interplay between these circuits may be an important mechanism in understanding the psychedelic effects.

This study has some limitations. Firstly, social cognition tasks were not directly assessed, to not interfere with the psychedelic experience, limiting insights into the effects of DMT on social cognition. Additionally, the inclusion of healthy experienced DMT users restricts the generalizability of the findings to other populations, such as naïve individuals or participants with mental health disorders. Notably, there exists evidence that individual baseline functional connectivity is connected to the subsequent alterations following psychedelic administration (Preller et al., 2020). Another limitation is the relatively small sample size although the within-subject design comparing active and control conditions still enhanced the statistical power. Additionally, while our study combines seed regions across both hemispheres to provide a comprehensive view of connectivity patterns, this approach may overlook potential lateralization effects. Further investigation is needed to understand the persistence of changes in resting-state connectivity beyond the acute phase, particularly with regard to its potential clinical significance.

## Data Availability

The raw data supporting the conclusions of this article will be made available by the authors, without undue reservation.
