# Supplementary material for: Increased functional connectivity between brain regions involved in social cognition, emotion and affective-value in psychedelic states induced by N,N-Dimethyltryptamine (DMT)
Source: Front Pharmacol. 2024 Oct 30;15:1454628. doi: 10.3389/fphar.2024.1454628 (PMC11558042; doi:10.3389/fphar.2024.1454628)
Supplement: Supplementary file 1 [file Table1.DOCX]

Supplementary Material

# Supplementary Tables

**Supplementary Table 1.** Hallucinogen Rating Scale (HRS) scores in the DMT and Control Conditions.

| **Mean (SD)** | **DMT** | **Control** | **F** | **η2** |
| --- | --- | --- | --- | --- |
| Somaesthesia | 0,94 (0,52) | 0,12 (0,13) | 28,03 *** | 0,74 |
| Affect | 1,41 (0,49) | 0,61 (0,37) | 27,28 *** | 0,73 |
| Perception | 1,44 (0,56) | 0,29 (0,31) | 78,64 *** | 0,89 |
| Cognition | 0,91 (0,68) | 0,19 (0,28) | 17,56 ** | 0,64 |
| Volition | 1,21 (0,43) | 0,74 (0,48) | 6,07 * | 0,38 |
| Intensity | 2,71 (0,66) | 0,22 (0,34) | 129,04 *** | 0,93 |
| Total | 8,61 (2,36) | 2,17 (1,17) | 89,71 *** | 0,90 |

*p < .05 **p < .01 ***p < .001

**Supplementary Table 2**. Significant correlations between changes in functional connectivity and psychedelic effects. Spearman's correlation (r) between the alterations in functional connectivity (∆r) and in the Hallucinogen Rating Scale (∆HRS) in the DMT versus Control conditions (N=11), significant at a threshold of p<0,05.

| **Brain Region**  **Connection** | **∆r** | **HRS Scale** | **∆HRS** | **Correlation (r)**  **∆r - ∆HRS** | **p value** |
| --- | --- | --- | --- | --- | --- |
| pSMG – PCG | 0,224 | Intensity | 2,495 | 0,697 | 0,017 |
| pSMG – Precuneus | 0,384 | Volition | 0,476 | 0,618 | 0,043 |

pSMG – posterior supramarginal gyrus; PCG – posterior cingulate gyrus
